# Supplementary material for: The emergence of modern zoogeographic regions in Asia examined through climate–dental trait association patterns
Source: Nat Commun. 2023 Dec 11;14:8194. doi: 10.1038/s41467-023-43807-w (PMC10713550; doi:10.1038/s41467-023-43807-w)
Supplement: Supplementary file 1 — Supplementary Information [file 41467_2023_43807_MOESM1_ESM.pdf]

Supplementary Information

The emergence of modern zoogeographic regions in Asia examined through climate–dental trait association patterns (Liu *et al.*)

Table 1 Data resources for the elevation estimates for the Tibetan Plateau from Figure 3.

Time interval	Site	Estimation method			
		Isotope O	Isotope C	Plant fossil	Vertebrate fossil
Early Miocene	Lunpola	4500–4900 m [1]	3000 m [2]	$<$ 3190 m [3]	3000 m [4]
	Hoh Xil	4200 m [1]		3000 m [5]	
Middle Miocene	Namling	5200 m [6]		4689 m [7]<br>$<$ 3000 m [8]	
early Late Miocene	Biru				$<$ 2500 m [9]
late Late Miocene	Gyirong	5850 m [10]	$<$ 2900 m [11]	$<$ 2900 m [9]	$<$ 2900 m [11]
Pliocene	Zanda	6000 m [12]			4000 m [13]

Table 2 Coordinates of the corners of the rectangles used to define the study region. The first five formed the dataset considered in our previous study [14] whereas the last four have been added in the present study and constitute the northern extension of our dataset.

Name	south-west	north-east
South East	10°N, 90°E	20°N, 115°E
South West	5°N, 66°E	28°N, 90°E
North East	20°N, 80°E	35°N, 125°E
North West	28°N, $67^\circ30'$E	$37^\circ30'$N, 90°E
North Mid	35°N, 80°E	40°N, 120°E
Extension North East	42°N, 130°E	50°N, 142°E
Extension North Mid	42°N, 125°E	50°N, 130°E
Extension Korea	30°N, 125°E	42°N, 130°E
Extension West	36°N, $67^\circ30'$E	50°N, 125°E

Table 3 Geographic conditions defining the groups of localities for computing and comparing dental traits and climate trends in Figure 3.

	x_1	x_2
(A)	28°N	
(B)	18°N, 106°E	36°N, 70°E
(C)	43°N, 124°E	24°N, 87°E

condition	group
north of (A)	northern Asia
south of (A)	southern Asia
north-east of (B) and south-east of (C)	northwestern (NW) China
north-east of (B) and north-west of (C)	southeastern (SE) China

References

[1] Polissar, P. J., Freeman, K. H., Rowley, D. B., McInerney, F. A. & Currie, B. S. Paleoaltimetry of the tibetan plateau from d/h ratios of lipid biomarkers. *Earth and Planetary Science Letters* **287** (1), 64–76 (2009). https://doi.org/10.1016/j.epsl.2009.07.037

[2] Jia, G., Bai, Y., Ma, Y., Sun, J. & Peng, P. Paleoelevation of tibetan lunpola basin in the oligocene–miocene transition estimated from leaf wax lipid dual isotopes. *Global and Planetary Change* **126**, 14–22 (2015). https://doi.org/10.1016/j.gloplacha.2014.12.007

[3] Sun, J. *et al.* Palynological evidence for the latest oligocene–early miocene paleoelevation estimate in the lunpola basin, central tibet. *Palaeogeography, Palaeoclimatology, Palaeoecology* **399**, 21–30 (2014). https://doi.org/10.1016/j.palaeo.2014.02.004

[4] Deng, T. *et al.* A mammalian fossil from the dingqing formation in the lunpola basin, northern tibet, and its relevance to age and paleo-altimetry. *Chinese Science Bulletin* **57** (2), 261–269 (2012). https://doi.org/10.1007/s11434-011-4773-8

[5] Sun, B. *et al.* Early miocene elevation in northern tibet estimated by palaeobotanical evidence. *Scientific Reports* **5** (1), 10379 (2015). https://doi.org/10.1038/srep10379

[6] Currie, B. S., Rowley, D. B. & Tabor, N. J. Middle Miocene paleoaltimetry of southern Tibet: Implications for the role of mantle thickening and delamination in the Himalayan orogen. *Geology* **33** (3), 181–184 (2005). https://doi.org/10.1130/G21170.1

[7] Spicer, R. A. *et al.* Constant elevation of southern tibet over the past 15 million years. *Nature* **421** (6923), 622–624 (2003). https://doi.org/10.1038/nature01356

[8] Zhou, Z., Yang, Q. & Xia, K. Fossils of quercus sect. heterobalanus can help explain the uplift of the himalayas. *Chinese Science Bulletin* **52** (2), 238–247 (2007). https://doi.org/10.1007/s11434-007-0005-7

[9] Deng, T. & Ding, L. Paleoaltimetry reconstructions of the tibetan plateau: progress and contradictions. *National Science Review* **2** (4), 417–437 (2015). https://doi.org/10.1093/nsr/nwv062

[10] Rowley, D. B., Pierrehumbert, R. T. & Currie, B. S. A new approach to stable isotope-based paleoaltimetry: implications for paleoaltimetry and paleohypsometry of the high himalaya since the late miocene. *Earth and Planetary Science Letters* **188** (1), 253–268 (2001). https://doi.org/10.1016/S0012-821X(01)00324-7

[11] Wang, Y., Deng, T. & Biasatti, D. Ancient diets indicate significant uplift of southern tibet after ca. 7 ma. *Geology* **34** (4), 309–312 (2006). https://doi.org/10.1130/G22254.1

[12] Saylor, J. E. *et al.* The late miocene through present paleoelevation history of southwestern tibet. *American Journal of Science* **309** (1), 1–42 (2009). https://doi.org/10.2475/01.2009.01

[13] Deng, T. *et al.* Locomotive implication of a pliocene three-toed horse skeleton from tibet and its paleo-altimetry significance. *Proceedings of the National Academy of Sciences* **109** (19), 7374–7378 (2012). https://doi.org/10.1073/pnas.1201052109

[14] Galbrun, E., Tang, H., Kaakinen, A. & Žliobaitė, I. Redescription mining for analyzing local limiting conditions: A case study on the biogeography of large mammals in china and southern asia. *Ecological Informatics* **63**, 101314 (2021). https://doi.org/10.1016/j.ecoinf.2021.101314
