# Supplementary material for: The emergence of modern zoogeographic regions in Asia examined through climate–dental trait association patterns
Source: Nat Commun. 2023 Dec 11;14:8194. doi: 10.1038/s41467-023-43807-w (PMC10713550; doi:10.1038/s41467-023-43807-w)
Supplement: Supplementary file 3 — Reporting Summary [file 41467_2023_43807_MOESM3_ESM.pdf]

nature portfolio

Corresponding author(s): Liu Liping
Galbrun Esther

Last updated by author(s): Nov 29, 2023

Reporting Summary

Nature Portfolio wishes to improve the reproducibility of the work that we publish. This form provides structure for consistency and transparency in reporting. For further information on Nature Portfolio policies, see our Editorial Policies and the Editorial Policy Checklist.

Statistics

For all statistical analyses, confirm that the following items are present in the figure legend, table legend, main text, or Methods section.

n/a	Confirmed	
☐	☒	The exact sample size (*n*) for each experimental group/condition, given as a discrete number and unit of measurement
☒	☐	A statement on whether measurements were taken from distinct samples or whether the same sample was measured repeatedly
☒	☐	The statistical test(s) used AND whether they are one- or two-sided<br>*Only common tests should be described solely by name; describe more complex techniques in the Methods section.*
☐	☒	A description of all covariates tested
☒	☐	A description of any assumptions or corrections, such as tests of normality and adjustment for multiple comparisons
☐	☒	A full description of the statistical parameters including central tendency (e.g. means) or other basic estimates (e.g. regression coefficient) AND variation (e.g. standard deviation) or associated estimates of uncertainty (e.g. confidence intervals)
☒	☐	For null hypothesis testing, the test statistic (e.g. *F*, *t*, *r*) with confidence intervals, effect sizes, degrees of freedom and *P* value noted<br>*Give P values as exact values whenever suitable.*
☒	☐	For Bayesian analysis, information on the choice of priors and Markov chain Monte Carlo settings
☒	☐	For hierarchical and complex designs, identification of the appropriate level for tests and full reporting of outcomes
☒	☐	Estimates of effect sizes (e.g. Cohen's *d*, Pearson's *r*), indicating how they were calculated

Our web collection on statistics for biologists contains articles on many of the points above.

Software and code

Policy information about availability of computer code

Data collection	To allow full reproducibility of our analysis, the scripts that fully automate the processing pipeline as described in the manuscript, along with a copy of the source data that we used, are available in (https://doi.org/10.5281/zenodo.10118849) [24].<br><br>For preparing paleoclimate model simulations<br>- Norwegian Earth System Model (NorESM, https://doi.org/10.1038/nature13705) [74]<br>- Community Climate System version 4 (CCSM4, https://doi.org/10.5194/gmd-6-549-2013) [75] with data provided by ecoClimate (https://doi.org/10.17161/bi.v10i0.4955, https://www.ecoclimate.org) [76]<br>- Earth System Modelling Framework (ESMF version 8.0.0, https://earthsystemmodeling.org, https://github.com/esmf-org/esmf/releases/tag/ESMF_8_0_0)<br>- dismo R package (version 1.1-4, https://cran.r-project.org/web/packages/dismo/)<br><br>[74] Zhang, Z. et al. Aridification of the sahara desert caused by tethys sea shrinkage during the late miocene. Nature 513 (7518), 401–404 (2014). https://doi.org/10.1038/nature13705<br>[75] Rosenbloom, N. A., Otto-Bliesner, B. L., Brady, E. C. & Lawrence, P. J. Simulating the mid-pliocene warm period with the CCSM4 model. Geoscientific Model Development 6 (2), 549–561 (2013).<br>[76] Lima-Ribeiro, M. S. et al. EcoClimate: a database of climate data from multiple models for past, present, and future for macroecologists and biogeographers. Biodiversity Informatics 10 (2015). https://doi.org/10.17161/bi.v10i0.4955
Data analysis	To allow full reproducibility of our analysis, the scripts that fully automate the processing pipeline as described in the manuscript, along with a copy of the source data that we used, are available in (https://doi.org/10.5281/zenodo.10118849) [24].

For mining redescriptions:
- Siren interface (https://doi.org/10.1145/3007212) [81], using the ReReMi algorithm (https://doi.org/10.1002/sam.11145) [82] (https://gitlab.inria.fr/egalbrun/siren, commit f154c53b)

[81] Galbrun, E. & Miettinen, P. Mining redescriptions with siren. ACM Transactions on Knowledge Discovery from Data (TKDD) 12 (1), 6:1–6:30 (2018). https://doi.org/10.1145/3007212
[82] Galbrun, E. & Miettinen, P. From black and white to full color: Extending redescription mining outside the boolean world. Statistical Analysis and Data Mining 5 (4), 284–303 (2012). https://doi.org/10.1002/sam.11145

For manuscripts utilizing custom algorithms or software that are central to the research but not yet described in published literature, software must be made available to editors and reviewers. We strongly encourage code deposition in a community repository (e.g. GitHub). See the Nature Portfolio guidelines for submitting code & software for further information.

Data

Policy information about availability of data

All manuscripts must include a data availability statement. This statement should provide the following information, where applicable:

- Accession codes, unique identifiers, or web links for publicly available datasets
- A description of any restrictions on data availability
- For clinical datasets or third party data, please ensure that the statement adheres to our policy

All prepared input data and results analyzed during the current study are available in (https://doi.org/10.5281/zenodo.10118849) [24], where they can also be visualized as maps.
Data sources:
Present-day climate: WorldClim2 (http://www.worldclim.com/version2) [73]
Present-day species occurrence: International Union for Conservation of Nature (IUCN, https://www.iucn.org/) processed by [18]
Fossil occurrence data: New and Old Worlds Database of Fossil Mammals (NOW, https://nowdatabase) [71]
[18] Oksanen, O., Žliobaitė, I., Saarinen, J., Lawing, A. M. & Fortelius, M. A humboldtian approach to life and climate of the geological past: Estimating palaeotemperature from dental traits of mammalian communities. Journal of Biogeography 46 (8), 1760–1776 (2019). https://doi.org/10.1111/jbi.13586
[71] The NOW Community. New and old worlds database of fossil mammals (NOW) (2023). https://doi.org/10.5281/zenodo.4268068, Licensed under CC BY 4.0
[73] Fick, S. E. & Hijmans, R. J. WorldClim 2: new 1-km spatial resolution climate surfaces for global land areas. International Journal of Climatology 37 (12), 4302–4315 (2017). https://doi.org/10.1002/joc.5086

Research involving human participants, their data, or biological material

Policy information about studies with human participants or human data. See also policy information about sex, gender (identity/presentation), and sexual orientation and race, ethnicity and racism.

Reporting on sex and gender	N/a
Reporting on race, ethnicity, or other socially relevant groupings	N/a
Population characteristics	N/a
Recruitment	N/a
Ethics oversight	N/a

Note that full information on the approval of the study protocol must also be provided in the manuscript.

Field-specific reporting

Please select the one below that is the best fit for your research. If you are not sure, read the appropriate sections before making your selection.

☐ Life sciences ☐ Behavioural & social sciences ☒ Ecological, evolutionary & environmental sciences

For a reference copy of the document with all sections, see nature.com/documents/nr-reporting-summary-flat.pdf

Life sciences study design

All studies must disclose on these points even when the disclosure is negative.

Sample size	N/a
Data exclusions	N/a

Replication	N/a
Randomization	N/a
Blinding	N/a

Behavioural & social sciences study design

All studies must disclose on these points even when the disclosure is negative.

Study description	N/a
Research sample	N/a
Sampling strategy	N/a
Data collection	N/a
Timing	N/a
Data exclusions	N/a
Non-participation	N/a
Randomization	N/a

Ecological, evolutionary & environmental sciences study design

All studies must disclose on these points even when the disclosure is negative.

Study description	We apply a computational data analysis technique, called redescription mining, to study the associations between the prevailing herbivore dental traits of mammalian communities and climatic conditions.<br>First, we extract redescriptions from present-day data, and select among the obtained results nine that have a high accuracy on that dataset and together provide a good coverage of the study area as well as of the different dental traits variables.<br>Next, we evaluate them on our dataset from the past, that combines data from the fossil record with paleoclimate model simulations, aiming to track the emergence of these modern zoogeographical regions during the Neogene.
Research sample	Our datasets contain a collection of localities, with variables recording the dental traits of mammalian communities, on one hand, and climatic conditions, on the other hand, for each locality.<br><br>Data sources:<br>Present-day climate: WorldClim2 (http://www.worldclim.com/version2) [73]<br>Present-day species occurrence: International Union for Conservation of Nature (IUCN, https://www.iucn.org/) processed by [18]<br>Fossil occurrence data: New and Old Worlds Database of Fossil Mammals (NOW, https://nowdatabase) [71]<br><br>[18] Oksanen, O., Žliobaitė, I., Saarinen, J., Lawing, A. M. & Fortelius, M. A humboldtian approach to life and climate of the geological past: Estimating palaeotemperature from dental traits of mammalian communities. Journal of Biogeography 46 (8), 1760–1776 (2019). https://doi.org/10.1111/jbi.13586<br>[71] The NOW Community. New and old worlds database of fossil mammals (NOW) (2023). https://doi.org/10.5281/zenodo.4268068, Licensed under CC BY 4.0<br>[73] Fick, S. E. & Hijmans, R. J. WorldClim 2: new 1-km spatial resolution climate surfaces for global land areas. International Journal of Climatology 37 (12), 4302–4315 (2017). https://doi.org/10.1002/joc.5086
Sampling strategy	The area studied in this work is focused on China, the Indian subcontinent and Southeast Asia.<br>The localities representing the present-day are square grid cells of 50x50 km across the study area.<br>The localities representing the past are fossil sites in the study area, spanning from the Early Miocene to the Pliocene (23–2.5 Ma).<br>We focus on large herbivorous mammals, selected by taxonomic orders, namely Perissodactyla, Artiodactyla, Primates, and Proboscidea and on dental traits, introduced and studied in previous work that are understood to have good correlations with the encountered environmental conditions,<br>including the climate.
Data collection	No new data was collected for the study.
Timing and spatial scale	The area studied in this work is focused on China, the Indian subcontinent and Southeast Asia. We use present-day data as well as fossil data spanning from the Early Miocene to the Pliocene (23–2.5 Ma).

Data exclusions	We exclude localities with fewer than three different taxa.
Reproducibility	Scripts for preparing the data and running the experiments are provided for reproducibility.
Randomization	N/a
Blinding	N/a

Did the study involve field work? ☐ Yes ☒ No

Field work, collection and transport

Field conditions	N/a
Location	N/a
Access & import/export	N/a
Disturbance	N/a

Reporting for specific materials, systems and methods

We require information from authors about some types of materials, experimental systems and methods used in many studies. Here, indicate whether each material, system or method listed is relevant to your study. If you are not sure if a list item applies to your research, read the appropriate section before selecting a response.

Materials & experimental systems

n/a	Involved in the study	
☒	☐	Antibodies
☒	☐	Eukaryotic cell lines
☒	☐	Palaeontology and archaeology
☒	☐	Animals and other organisms
☒	☐	Clinical data
☒	☐	Dual use research of concern
☒	☐	Plants

Methods

n/a	Involved in the study	
☒	☐	ChIP-seq
☒	☐	Flow cytometry
☒	☐	MRI-based neuroimaging

Antibodies

Antibodies used	N/a
Validation	N/a

Eukaryotic cell lines

Policy information about cell lines and Sex and Gender in Research

Cell line source(s)	N/a
Authentication	N/a
Mycoplasma contamination	N/a
Commonly misidentified lines (See ICLAC register)	N/a

Palaeontology and Archaeology

Specimen provenance	No specimen level data was used.

Specimen deposition	N/a
Dating methods	N/a

☐ Tick this box to confirm that the raw and calibrated dates are available in the paper or in Supplementary Information.

Ethics oversight	N/a

Note that full information on the approval of the study protocol must also be provided in the manuscript.

Animals and other research organisms

Policy information about studies involving animals; ARRIVE guidelines recommended for reporting animal research, and Sex and Gender in Research

Laboratory animals	N/a
Wild animals	N/a
Reporting on sex	N/a
Field-collected samples	N/a
Ethics oversight	N/a

Note that full information on the approval of the study protocol must also be provided in the manuscript.

Clinical data

Policy information about clinical studies

All manuscripts should comply with the ICMJEguidelines for publication of clinical research and a completedCONSORT checklist must be included with all submissions.

Clinical trial registration	N/a
Study protocol	N/a
Data collection	N/a
Outcomes	N/a

Dual use research of concern

Policy information about dual use research of concern

Hazards

Could the accidental, deliberate or reckless misuse of agents or technologies generated in the work, or the application of information presented in the manuscript, pose a threat to:

No	Yes	
☒	☐	Public health
☒	☐	National security
☒	☐	Crops and/or livestock
☒	☐	Ecosystems
☒	☐	Any other significant area

Experiments of concern

Does the work involve any of these experiments of concern:

No	Yes	
☒	☐	Demonstrate how to render a vaccine ineffective
☒	☐	Confer resistance to therapeutically useful antibiotics or antiviral agents
☒	☐	Enhance the virulence of a pathogen or render a nonpathogen virulent
☒	☐	Increase transmissibility of a pathogen
☒	☐	Alter the host range of a pathogen
☒	☐	Enable evasion of diagnostic/detection modalities
☒	☐	Enable the weaponization of a biological agent or toxin
☒	☐	Any other potentially harmful combination of experiments and agents

Plants

Seed stocks: N/a

Novel plant genotypes: N/a

Authentication: N/a

ChIP-seq

Data deposition

☐ Confirm that both raw and final processed data have been deposited in a public database such as GEO.

☐ Confirm that you have deposited or provided access to graph files (e.g. BED files) for the called peaks.

Data access links
May remain private before publication.: N/a

Files in database submission: N/a

Genome browser session
(e.g. UCSC): N/a

Methodology

Replicates: N/a

Sequencing depth: N/a

Antibodies: N/a

Peak calling parameters: N/a

Data quality: N/a

Software: N/a

Flow Cytometry

Plots

Confirm that:

☐ The axis labels state the marker and fluorochrome used (e.g. CD4-FITC).

☐ The axis scales are clearly visible. Include numbers along axes only for bottom left plot of group (a 'group' is an analysis of identical markers).

☐ All plots are contour plots with outliers or pseudocolor plots.

☐ A numerical value for number of cells or percentage (with statistics) is provided.

Methodology

Sample preparation	N/a
Instrument	N/a
Software	N/a
Cell population abundance	N/a
Gating strategy	N/a

☐ Tick this box to confirm that a figure exemplifying the gating strategy is provided in the Supplementary Information.

Magnetic resonance imaging

Experimental design

Design type	N/a
Design specifications	N/a
Behavioral performance measures	N/a

Acquisition

Imaging type(s)	N/a
Field strength	N/a
Sequence & imaging parameters	N/a
Area of acquisition	N/a

Diffusion MRI ☐ Used ☒ Not used

Preprocessing

Preprocessing software	N/a
Normalization	N/a
Normalization template	N/a
Noise and artifact removal	N/a
Volume censoring	N/a

Statistical modeling & inference

Model type and settings	N/a
Effect(s) tested	N/a

Specify type of analysis: ☐ Whole brain ☐ ROI-based ☐ Both

Statistic type for inference (See Eklund et al. 2016)	N/a
Correction	N/a

Models & analysis

n/a	Involved in the study
☒	☐ Functional and/or effective connectivity
☒	☐ Graph analysis
☒	☐ Multivariate modeling or predictive analysis

Functional and/or effective connectivity	N/a
Graph analysis	N/a
Multivariate modeling and predictive analysis	N/a
